# Evaluation of antibacterial and toxicity activity *in vitro *of extracts from *Tournefortia bicolor *S.W (Boraginaceae)

**DOI:** 10.1186/1753-6561-8-S4-P31

**Published:** 2014-10-01

**Authors:** Isabelle Souza de Mélo Silva, Andressa Letícia Lopes da Silva, Raíssa Fernanda Evangelista Pires dos Santos, Renata Rego de Souza, Andriele Mendonça Barbosa, Klebson Silva Santos, Mariene Ribeiro Amorim, Lenilson Santos da Trindade, Laiza Canielas Krause, Eliane Aparecida Campesatto, Francine Ferreira Padilha, Maria Lysete de Assis Bastos

**Affiliations:** 1 Instituto de Tecnologia e Pesquisa, Universidade Tiradentes - Av. Murilo Dantas, 300, Farolândia - Aracaju, Sergipe, CEP 49032-490, Brazil; 2 Laboratório de Pesquisa em Tratamento de Feridas, Universidade Federal de Alagoas, Campus A. C. Simões - Av. Lourival Melo Mota, s/n, Cidade Universitária - Maceió, Alagoas, CEP 57072-900, Brazil; 3 Laboratório de Farmacologia e Imunidade, Universidade Federal de Alagoas, Campus A. C. Simões - Av. Lourival Melo Mota, s/n, Cidade Universitária - Maceió, Alagoas, CEP 57072-900, Brazil

## Background

The reported use of medicinal plants has shown that they are part of human evolution and were the first therapeutic resources used by people. The search for new alternatives to replace or serve as an adjunct to antimicrobial treatment available has been prioritized. Brazil has a great biodiversity of plants, which are popularly used for medicinal form. Vegetables have been widely used in health care due to its medicinal properties, such as antibacterial, antifungal and immunomodulatory activities [1], but can be evaluated for their antitumor potential, insecticide, pesticide, or even cercaricide among other biological actions that depend on cytotoxicity [2,3]. The aim of this study was to evaluate the *in vitro *antibacterial activity of the extract, fractions and subfractions of a native plant to the state of Alagoas, the *Tournefortia bicolor*.

## Methods

Experimental *in vitro *study, conducted at the Research Laboratory of Wound Care, at Federal University of Alagoas. Three samples were tested at *T. bicolor *(Sheet Part - Neutral hexane; Stem Part - Acetate fraction; Stem Part - Chloroform fraction and methanol fraction). The samples were subjected to bioassays in Petri dishes by the disk diffusion method to evaluate the potential for bacterial growth inhibition by halo formation facing the strains of *Staphylococcus aureus *ATCC 25923, *Klebsiella pneumoniae *ATCC 31488, *Shigella flexneri *ATCC 12022, *Enteroccocus faecalis *ATCC 29212, *Salmonella typhimurium *ATCC 14028, *Escherichia coli *ATCC 14942, *Pseudomonas aeruginosa *ATCC 27853 and *Streptococcus pyogenes *ATCC 12344, distributed by the American Type Cell Collection. All extracts were tested against *Brine Shrimps*.

## Results and conclusions

All samples suggested inactive against the strains tested, due to non-appearance of inhibition zone. Thus, due to inactivity of the samples in the disk diffusion test front of the strains bacterial tested, did not occur to testing of Minimum Inhibitory Concentration (MIC). The cytotoxicity avaliation of the samples was performed using the lethality assay against *Brine Shrimps*. The extracts evaluated, two had - if nontoxic. In toxicity tests with brine, only the hexane extract neutral - and leaf ethyl acetate - stems were presented nontoxic, with a mortality rate of 0% and 10%, respectively. The chloroform methanol extract - stem proved toxic with a mortality rate of 73.34%. All extracts were thymol as a positive control, which has the ability to break the cytoplasmic membrane and therefore carry the larvae of *Brine Shrimps *to death by dehydration [4], with 100% mortality in the *brine*. However extracts and pure compounds from plant species that have CL_50_<500 mg/mL are considered toxic, but can be evaluated for their antitumor potential, insecticide, pesticide, or even cercaricide to combat the infectious form of *Schistosoma mansoni *among other biological actions that depend on cytotoxicity [2,3]. Studies conducted at the National Cancer Institute in the United States, Brine correlated to high toxicity, or CL_50 _low concentrations with significant growth inhibition *in vitro *of cell lines derived from solid tumors [5].
